# Biodiversity influences the effects of oil disturbance on coastal ecosystems

**DOI:** 10.1002/ece3.8532

**Published:** 2022-01-24

**Authors:** Robyn A. Zerebecki, Kenneth L. Heck, John F. Valentine

**Affiliations:** ^1^ Dauphin Island Sea Lab Dauphin Island Alabama USA; ^2^ Present address: University of Louisiana Lafayette Louisina USA

**Keywords:** *Deepwater Horizon*, diversity‐stability, genetic diversity, oiling, species diversity

## Abstract

Biodiversity can enhance the response of ecosystems to disturbance. However, whether diversity can reduce the ecological effect of human‐induced novel and extreme disturbances is unclear. In April 2010, the *Deepwater Horizon* (*DwH*) platform exploded, allowing an uncontrolled release of crude oil into the northern Gulf of Mexico. Initial surveys following the spill found that ecological impacts on coastal ecosystems varied greatly across habitat‐type and trophic group; however, to date, few studies have tested the influence of local biodiversity on these responses. We used a meta‐analytic approach to synthesize the results of 5 mesocosm studies that included 10 independent oil experiments and 5 independent oil + dispersant experiments. We tested whether biodiversity increased the resistance and/or resilience of coastal ecosystems to oil disturbance and whether a biodiversity effect depended on the type of diversity present (taxonomic or genetic) and/or the response type measured (population, community, or ecosystem level). We found that diversity can influence the effects of oiling, but the direction and magnitude of this diversity effect varied. Diversity reduced the negative impact of oiling for within‐trophic‐level responses and tended to be stronger for taxonomic than genetic diversity. Further, diversity effects were largely driven by the presence of highly resistant or quick to recover taxa and genotypes, consistent with the insurance hypothesis. However, we found no effect of diversity on the response to the combination of oil and dispersant exposure. We conclude that areas of low biodiversity may be particularly vulnerable to future oil disturbances and provide insight into the benefit of incorporating multiple measures of diversity in restoration projects and management decisions.

## Graphical abstract

Oil spill impacts on marine ecosystems can vary greatly: from negligible to total destruction. The extent of impacts depends on a variety of factors including oil properties and habitat characteristics; yet, whether local diversity can also enhance resistance and resilience is unclear. Using a meta‐analysis, we found that diversity can reduce the negative effects of oiling, but this diversity effect was primarily evident for within‐trophic‐level responses, and stronger for taxonomic than genetic diversity. Together, highlighting that conserving and maintaining biodiversity may provide a benefit to marine ecosystems in the face of future perturbations.

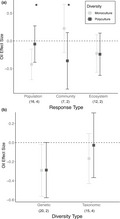

## 1 INTRODUCTION

There is substantial evidence that biodiversity can enhance ecosystem stability in response to perturbations (Cardinale et al., 2012; Loreau & de Mazancourt, 2013; Tilman et al., 2014). This biodiversity effect can be underpinned by numerous, nonexclusive mechanisms (see Lehman & Tilman, 2000; Loreau & de Mazancourt, 2013; de Mazancourt et al., 2013; Tilman et al., 2006). For instance, because species can vary in both their resistance to, and rate of recovery from, disturbance (Loreau & de Mazancourt, 2013; Reice, 1994), more diverse communities have a greater likelihood of including these more disturbance tolerant or quicker to recover species that can compensate for the loss of those less tolerant (i.e., the insurance hypothesis; Tilman, 1996; Yachi & Loreau, 1999). Alternatively, more diverse systems often contain species with increased trait and functional variation that can limit the severity of disturbance effects through complementarity (e.g., niche partitioning) or facilitation (Hughes et al., 2007; Mulder et al. 2001). However, with anthropogenic impacts increasing both the frequency and magnitude of disturbance (e.g., drought, temperature; Coumou & Rahmstord, 2012; Easterling et al., 2000; Meehl & Tebaldi, 2004) and creating novel perturbations (e.g., habitat alteration, oil spills; Halpern et al., 2008; Vitousek et al., 1997), it is unclear whether the ecological impacts of these novel and extreme events can be reduced by greater biodiversity.

In April 2010, the *Deepwater Horizon* (DwH) platform exploded, and the subsequent destruction of the blowout preventer led to the uncontrolled release of over 3 million barrels of crude oil into the northern Gulf of Mexico (Barron et al., 2020; Beyer et al., 2016; Michel et al., 2013). In response to this spill, several management actions were implemented to reduce the amount of oil that reached shore, including the application of chemical dispersants, burning of oil slicks, and enhanced freshwater release from river diversions (Beyer et al., 2016; Grabowski et al., 2017). Some of these techniques can also have detrimental ecological impacts that are similar to or greater than oil exposure (Beyer et al., 2016). For example, prior studies have shown that chemically dispersed oil can have more deleterious effects than oiling‐only in some plankton species (Almeda et al., 2014; Beyer et al., 2016) and oysters (Laramore et al., 2014; Vignier et al., 2015). Ultimately, more than 2000 km of shoreline was oiled (Barron et al., 2020; Beyer et al., 2016), impacting a multitude of nearshore, interconnected ecosystems that included wetlands, seagrass beds, and oyster reefs (Baker et al., 2017).

After the *DwH* disaster, scientists have evaluated the impact of oil and other spill‐related environmental interventions (e.g., dispersant application, freshwater input) to assess the direct impacts on the physiology, behavior, fitness, and population dynamics of resident species, as well as the cascading effects on community and ecosystem functions in these systems (see Andersen, 2014; Fodrie et al., 2014; Henkel et al., 2012; Mendelssohn et al., 2012; Powers et al., 2017a; Rabalais & Turner, 2016). Field studies demonstrated that the impacts of, and recovery from, DwH oiling varied greatly in magnitude across the region, ranging from total losses of habitat and associated species, to negligible effects (Andersen, 2014; Fleeger et al., 2015, 2019; Fodrie et al., 2014; Lin & Mendelssohn, 2012; Martin et al., 2020; Pennings et al., 2014; Powers et al., 2017b; Rabalais & Turner, 2016; Silliman et al., 2012). This variability can depend on the amount of oiling and the heterogeneous properties of the oil's toxicity, exposure duration, and clean‐up methods (Michel & Rutherford, 2014; Zengel et al., 2015), as well as physical factors such as shoreline orientation and tidal regime (Lin et al., 2016; Mendelssohn et al., 2012; Michel & Rutherford, 2014; Powers et al., 2017b). Yet, the susceptibility and tolerance to oiling also varies among and within species inhabiting these nearshore ecosystems (for plants, see Hughes et al., 2018; Lin et al., 2016; Pezeshki et al., 2000; for aquatic invertebrates, Fleeger et al., 2015, 2019; for insects, Pennings et al., 2014; and for fishes, Fodrie et al., 2014), likely due to variation in behavioral or physiological traits. Given the variability in species’ responses to oil, species composition and/or biodiversity likely also affected the realized impact of oiling. However, we currently have little understanding of how biodiversity affects the response of coastal ecosystems to oiling.

Greater diversity of marine organisms can mitigate the negative effects of other types of disturbance (Salo & Gutstafsson, 2016; Stachowicz et al., 2007; Worm et al., 2006), suggesting that diversity may also reduce the negative effects of oiling in nearshore habitats. For instance, both taxonomic and genetic diversity can increase resilience and resistance to physical stress and herbivory (Cardinale et al., 2012; Hughes & Stachowicz, 2004; Reusch et al., 2005; Tilman et al., 2014). Because common nearshore ecosystems exposed to *DwH* oiling are dominated by single foundation species (e.g., oyster reefs, salt marshes), genetic diversity may be particularly important for reducing the negative effects of disturbance in these ecosystems (Hughes et al., 2008). To our knowledge, no study has evaluated whether the type of diversity influences the biodiversity–stability relationship as it pertains to oil exposure. Previous studies have also shown that the strength of diversity effects varies across trophic levels, depending on both the level at which diversity is manipulated and at which responses are measured (Balvanera et al., 2006; Bailey et al., 2009; Gamfeldt et al., 2015; Stachowciz et al., 2007; Whitlock, 2014). To date, most marine studies on diversity–stability have focused on within‐trophic‐level responses (Stachowicz et al., 2007) from primary producers (Allison, 2004; Boyer et al., 2009; Corcoran & Boeing, 2012; Hughes & Stachowicz, 2004) to invertebrates (Emmerson et al., 2001; Waldbusser et al., 2004) and fishes (Duffy et al., 2016; Nash et al., 2016), likely due to the ease of making straightforward predictions for population‐level responses. Diversity should have a positive effect if disturbance is selective, such that taxa or genotypes vary in their responses. However, if all species or genotypes respond similarly, diversity would be expected to have a limited effect (Allison, 2004). In contrast, multiple predictions are possible when examining diversity effects on the response to disturbance across trophic levels (e.g., community‐ and ecosystem‐level effects) due to the complexity of interactions (Rooney & McCann, 2012; Thibault & Loreau, 2005).

Here, we synthesize multiple independent experiments that tested whether taxonomic and genetic diversity influenced the response of nearshore coastal ecosystems in the northern Gulf of Mexico to oil and chemical dispersant exposure. The variation in both the experimental duration and sampling timing among experiments (see Table 1) meant that some studies measured resistance (i.e., the degree of impact), and while others measured resilience (e.g., resistance + recovery; Hughes et al., 2007, Griffin et al., 2009, Oliver et al., 2015). Thus, we assessed how diversity influenced the response to disturbance, which included both resistance and resilience. Specifically, we tested the hypothesis that the wide variation in response to oiling found in the *DwH* literature could be the result of variation in biodiversity across sites/habitats. We evaluated mesocosm experiments that manipulated both diversity and oil exposure to explore how biodiversity affects the response to oiling in coastal ecosystems across levels of biological organization (population‐, community‐, or ecosystem‐level processes) or type of biodiversity (i.e., taxonomic and genetic). Because we observed significant diversity effects, we also assessed whether the best monoculture outperformed average polyculture mixtures in each experiment (i.e., transgressive overyielding) to detect the potential underlying mechanism of these diversity effects. For instance, in the absence of transgressive overyielding, diversity effects are likely driven by the inclusion of certain taxa and genotypes that are more capable of withstanding and/or recovering from oil exposure than others (i.e., identity effect), whereas observations of transgressive overyielding are suggestive that complementarity effects (e.g., facilitation) are also important. We expect that our findings will aid in making accurate predictions about the role of diversity in coastal habitat vulnerability to future oil spills and that this will be useful for future management decisions and restoration practices.

**TABLE 1:** Overview of conducted ACER subgroups mesocosm studies

Experimental ecosystem	Sub‐groups	Organism	Studies	Duration (# Days)	Other treatments	Total # experiments	Diversity category	# of Polyculture richness levels
Subtidal Oyster Reef	Oyster^a^	Eastern oyster (*Crassostrea viriginica*)	1	21	Salinity (2 levels); Dispersant Presence/Absence	4	Genetic	2 (2 Parental pairs, **3 Parental pairs**)
Coastal Wetland	Wetlands^b^ & Nitrogen	Smooth Cordgrass *(Spartina alterniflora*) & Black mangrove (*Avicennia germinans)*	1		Diversity (Taxonomic & Genetic)	3*	Genetic	1 (**3 Genotypes**)
				365				
							Taxon	1 (**2 Species**)
Nearshore Subtidal (Seagrass Bed, Oyster Reef, Marsh) Community	Consumer (Fishes)	Hardhead catfish (*Ariopsis felis*), Gulf killifish (*Fundulus grandis*), Gulf toadfish (*Opsanus beta*), and adult Blue crabs (*Callinectes sapidus*)	1	2	NA	1	Taxon	2 (2 Predator species, **4 Predator species)**
Epipelagic Plankton Community	Phytoplankton	Diatom (*Skeletonema* sp.), Dinoflagellate (*Prorocentrum* sp.) and Chlorophyte (*Tetraselmis* sp.) algae	1	6–21	Oil Concentrations (3 levels); Dispersant Presence/Absence +Concentrations (3 levels)	6	Taxon	1 (**3 Species**)
Benthic Mudflat	Infauna^c^	Polychaete *(Owenia fusiformis)* & Brittlestar (*Hemipholia elognata)*	1	25	Density (Varied in Monocultures; not incorporated)	1	Taxon	1 (**2 Species**)

Note

Experimental system is the ecosystem mimicked. Organisms described are the taxa used in diversity treatments. Studies indicated the total number that many met our meta‐analysis inclusion criteria. Duration is the length of time each study was conducted. Other treatments were included as independent experiments in our analysis; total experiment includes both oil and oil + dispersant experiments. *Within the wetland group, two independent experiment (*Spartina* only treatments vs. *Spartina* + *Avicennia*) for genetic diversity, whereas comparison of taxonomic diversity included both genetic treatments. Polyculture richness levels manipulated in each experiment with those bold used in our analysis when there were multiple levels tested. Published papers from each subgroup denoted with footnotes.

^a^ Schrandt et al. (2018).

^b^ Hughes et al. (2018).

^c^ Dorgan et al. (2020).

## 2 METHODS

### 2.1 Study selection

To assess whether biodiversity can increase the resistance and resilience of coastal ecosystems of the northern Gulf of Mexico to oil disturbance, we conducted a meta‐analysis of mesocosm experiments conducted by the Alabama Center for Ecological Resilience (ACER). ACER consisted of 7 subgroups that studied oil and dispersant impacts on a range of coastal habitats and taxa (microbes to fishes; see Table 1). We gathered relevant data from the GRIIDC database (https://data.gulfresearchinitiative.org) using the keyword ACER and/or obtained experimental data directly from each subgroup. Together, the ACER subgroups conducted over 10 laboratory mesocosm studies on *DwH* oiling impacts in coastal ecosystems. However, to be included in our meta‐analysis, studies had to meet two criteria: (1) they had to manipulate both biodiversity (genetic and/or taxonomic diversity) and oil exposure and include a no‐oil control. If the study included additional treatments (e.g., variation in salinity, dispersant presence/absence), tested various levels of oil or dispersant concentrations, or manipulated multiple types of biodiversity, then we treated each additional treatment as an independent experiment. Because only one study included an independent dispersant treatment, we did not include dispersant only effects in our analysis; and (2) all individual species, genotypes, or family lines were required to be present in both monoculture and at least one polyculture mixture. If individual taxa were not replicated across diversity treatments, replicates that included these individuals were excluded from effect size calculations. For example, in phytoplankton studies, *Asterionellopsis sp*. was grown in monoculture but not polycultures; thus, we did not include *Asterionellopsis sp*. in monoculture averages. Our final dataset consisted of 5 studies that reported results of 10 independent oil‐only experiments and five independent oil + dispersant experiments (Tables 1 and 2). We evaluated the oil effects and oil +dispersant effects separately.

**TABLE 2:** Total number of experiments included both oil‐only (*n* = 10) and oil + dispersant experiments (*n* = 5)

Sub‐groups	Oil‐only Exp.	Oil + Dispersant Exp.	# of Responses	Measured responses		
				Population	Community	Ecosystem
Oyster	2 (2)	2 (2)	1	Shell height growth	NA	NA
Wetlands & Nitrogen	3* (22)	NA	Genetic (12)**	A. *Spartina* belowground biomass, *Spartina* leaf growth, Flower production, Seed production	B. Nitrite reductase (denitrifer) microbial Abundance, Nitrous oxide reductase (denitrifer) microbial Abundance, Chlorophyll a concentration, Bacterial Shannon Diversity	A. Denitrification Potential Rate, N2 fixation potential; B. Sediment oxygen demand, Phosphate flux
			Taxonomic (4)	Total Aboveground Biomass, total belowground biomass	NA	Denitrification Potential Rate, N2 fixation potential
Consumer (Fishes)	1 (3)	NA	3	NA	Prey Consumption Rate: juvenile Blue crab, Shrimp (*Palaemonetes* sp.), Fish (*Fundulus xenicus*) each individually	NA
Phytoplankton	3 (3)	3 (3)	1	Maximum Algal Growth Rate	NA	NA
Infauna	1 (5)	NA	5	Survival	NA	Sediment Oxygen Demand, & Bioturbation: Max. luminophore depth, Horizontal luminophore dispersion, and Luminophore subduction
Total	10 (35)	5 (5)				

Note

Parentheses denote the number of effect sizes (*k*) for both monoculture and polyculture in total within oil and oil + dispersant experiments. Responses were categorized into 3 levels of biological organization: Population: fitness and production metrics of target taxa or genotype manipulated in diversity treatment; Community: response of associated species in response to changes in target diversity; or Ecosystem: change in ecosystem functions or processes in response to variation in target diversity. Number of each response types in brackets * Within wetland group, 2 responses were included in each independent study (underlined). **For genetic diversity: A) were measured across independent taxonomic diversity experiments (6 responses x 2 diversity experiments), whereas B) only within taxonomic polycultures (6 responses).

For each study, we compiled data on the type of biodiversity manipulated (i.e., taxonomic or genetic), number of polyculture richness levels tested, levels and concentration of oil (or oil + dispersant) exposure, method of oil exposure (e.g., press vs. pulse, weathered or not, etc.), any additional treatment factors, responses variables measured, and the location, timing, and duration of the experiment. Studies had a limited range of polyculture richness levels (2–4), but were within the natural range of diversity in these systems. However, coupled with the low replication of richness levels among experiments, our ability to examine how the biodiversity–disturbance relationship changed across a richness gradient was restricted. Instead, we focused solely on a categorical comparison of monoculture and polyculture effects. As biodiversity effects often exhibit a nonlinear, saturating response (Cardinale et al., 2012), the most striking diversity effects tend to occur between the minimal and maximal levels of richness tested; thus, we used the highest richness level manipulated in each experiment to determine polyculture effect size. This also limits the over‐representation of individual studies and allows for equal number of monoculture and polyculture effect sizes in our models. To explore diversity effects on oiling impacts across a range of biological organizations, we also categorized every response variable from each experiment into one of 3 hierarchical categories: population‐, community‐, or ecosystem‐level effects or functions (see Table 2).

Many of these experiments measured multiple response variables (see Table 2) as well as responses at multiple time points; therefore, we had to account for this nonindependence within studies. To do so, we first limited the time points and responses included (see Appendix S1 for selection and inclusion details). Second, because we were interested in how response type mediated the effect of diversity on oil impacts, we accounted for the nonindependence of multiple response variables by including a random effect of experiment and independent study in our statistical model (see below). In total, we had 35 estimates of oil effect sizes and 5 estimates of oil +dispersant effect sizes for both monoculture and polyculture treatments (see Table 2, and Figures S1 and S2 for forest plots of individual effect sizes).

### 2.2 Calculation of oil or oil + Dispersant effect sizes

For each individual response variable, we calculated the effect size of oiling and its variance for both monoculture (d_mono_) and polyculture (d_poly_) treatments separately. For most studies, we used the raw data deposited in GRIIDC for each response variable. However, in a few instances, we used calculations to either estimate biomass (by allometric relationships) or growth rate (by logistic regression) or to account for controls (see Appendix S1 for more details). We used Hedges’ d as our measure of effect size, because using a standardized mean difference accounts for differences in the scale of individual response variables among studies, and because several individual responses within studies had magnitudes that differed in sign between oil treatments (Korciheva et al., 2013). We calculated Hedge's *d* oiling effect as follows:

$$d=\frac{\mu_{\text{oil}}‐\mu_{\text{non ‐ oil}\;}}{\sqrt{\frac{\left(n_{\text{oil}}‐1\right)s_{\text{oil}}^{2}+\left(n_{\text{non ‐ oil}}‐1\right)s_{\text{non ‐ oil}}^{2}}{n_{\text{oil}}+n_{\text{non ‐ oil}}‐2}}}\times J$$

where *µ* is the mean, *n* is the sample size and s is the standard deviation of either oiled or non‐oiled treatment. We also included a correction factor for small sample sizes, *J*, in all Hedge's *d* calculations as sample size ranged from 5 to 28.

$J=1‐\frac{3}{4\left(n_{\text{oil}}+n_{\text{non ‐ oil}}‐2\right)‐1}$

A positive *d* indicates that oiling increased performance, while a negative *d* indicates that oiling reduced performance. A Hedge's *d* with 95% confidence intervals encompassing zero indicate no difference between oiling and non‐oiling treatments. To conduct weighted analysis that incorporated a measure of precision for each individual study, we calculated the variance of each individual effect size as follows:

$$\nu_{d}=\frac{n_{\text{oil}}+n_{\text{non ‐ oil}\;}}{n_{\text{oil}}n_{\text{non ‐ oil}}}+\frac{d^{2}}{2\left(n_{\text{oil}}+n_{\text{non ‐ oil}}\right)}$$

and included the inverse of this variance as a weighting term in our statistical analysis. We used this same format when evaluating the combined effects of oil and dispersant.

Our full dataset, as detailed above, focused on the average effects of monocultures and polycultures on oiling effect sizes. However, because individual taxa or genotypes can vary in their response to disturbance, it can also be informative to determine if the average polyculture outperforms the best monoculture (i.e., transgressive overyielding). Observing a lack of transgressive overyielding provides evidence that the inclusion of specific taxa or genotype (identity effects) contributes to the observed diversity effect (Loreau & Mazancourt, 2013; O’Connor & Byrnes, 2014). In our case, we assessed the “best” monoculture as the monoculture for which taxa or genotypes were least affected by oiling. To accomplish this comparison, we removed any experiment or response that did not replicate individual monocultures, resulting in 28 oil‐only effect size estimates for each level of biodiversity. There was no reduction in the number of oil + dispersant effect sizes as all experiments had replicates of individual monoculture treatments.

### 2.3 Statistical analysis

We conducted a random effects meta‐analysis to test for the independent effects of biodiversity, response type, and diversity category on oiling impacts using two complementary methods. All analyses were conducted in R (version 3.5). First, we performed a multilevel meta‐regression using the meta (Balduzzi et al., 2019) and metafor (Viechtbauer, 2010) packages fit with a restricted maximum‐likelihood estimation. We also used these R packages to estimate heterogeneity via Higgins and Thompson’s (2002) *I* ^2^ using the metacont function and tested for data availability bias via funnel plots and Egger's regression test for funnel plot asymmetry (Egger et al., 1997). Egger's tests were conducted by modifying each model to include the standard error of the effect size as a moderator (following Habeck & Schultz, 2015). Second, we conducted a linear mixed model using the lme4 (Bates et al., 2015) and lmerTest (Kuznetsova et al., 2017) packages that used Satterthwaite approximation for degrees of freedom to generate *F* and *p*‐values. We applied both analyses on oil and oil + dispersant effect sizes separately. As linear mixed models and multilevel meta‐regression illustrated similar patterns, we describe the meta‐regression model in the main text and linear mixed model in the supplemental material (Appendix S2).

To assess whether oil effects varied as a function of biodiversity (2 levels: monoculture or polyculture), manipulated diversity category (2 levels: genetic or taxonomic) and response type (3 levels: population, community, or ecosystem), we employed a model that included biodiversity, the interaction between biodiversity and diversity category as well as the interaction between biodiversity and response type as fixed effects, and the inverse variance as a weighting factor.

$$\begin{array}{c} \text{Effect Size}\;∼\;\text{Biodiversity}\;+\;\text{Biodiversity: Response Type}\;+\\ \text{Biodiversity: Diversity Category}\;+\;\left(1|\;\text{Study}\right)\;+\;\left(1|\;\text{Experiment}/\text{Response}\;\#\right)\;+\;\left(1/\text{Variance}\right) \end{array}$$

We used this approach because we were only interested in whether diversity category and response type altered the magnitude and direction of biodiversity effect on oil impacts. We also included study (subgroup) identity and response variable nested in an experiment as random effects to account for the nonindependence of multiple responses within each experiment and variation among subgroups. To test oil + dispersant effects, we used a similar model (including weighting and random effects); however, we only included biodiversity as a fixed effect because with only two independent studies, we unable to assess either diversity category or response type as they were confounded with study identity.

We compared the explained heterogeneity *Q* statistic (*Q*_m_) using a Wald‐type chi‐square to test for significance of each independent and interactive factor. For any significant interaction, we used linear contrasts to assess monoculture versus polyculture response within other predictor factors (i.e., within individual levels of either diversity category or response type factor) using the glht function in the multcomp package and included a “holm” correction factor for multiple comparisons. An estimated mean effect size of each independent and interactive factor was calculated separately using the metafor package by removing the intercept from each statistical model. We also estimated the overall mean oil and oil + dispersant effect coefficients using a model that only contained the random effects.

## 3 RESULTS

### 3.1 Data set diagnostics

Our initial oil dataset had substantial heterogeneity (*I* ^2^ = 72.6%; Higgins & Thompson, 2002). To deal with this high heterogeneity, we were able to identify an outlier response in the oyster study and replaced it with a correlated response (See Appendix S1 for more details). This greatly reduced the heterogeneity (*I* ^2^ = 42.2%) of our dataset. Our oil + dispersant dataset also had high heterogeneity (*I* ^2^ = 53.1%). Funnel plots did not suggest availability bias within either oil or oil + dispersant datasets (see Figure S3). However, Egger's test detected availability bias in 1 dataset (oil effect size across average diversity treatments; *p* = .01), while our other 3 datasets showed no significant regression (*p* > .17).

### 3.2 Average monoculture and polyculture comparison

#### 3.2.1 A. Oil effects

**FIGURE 1:**
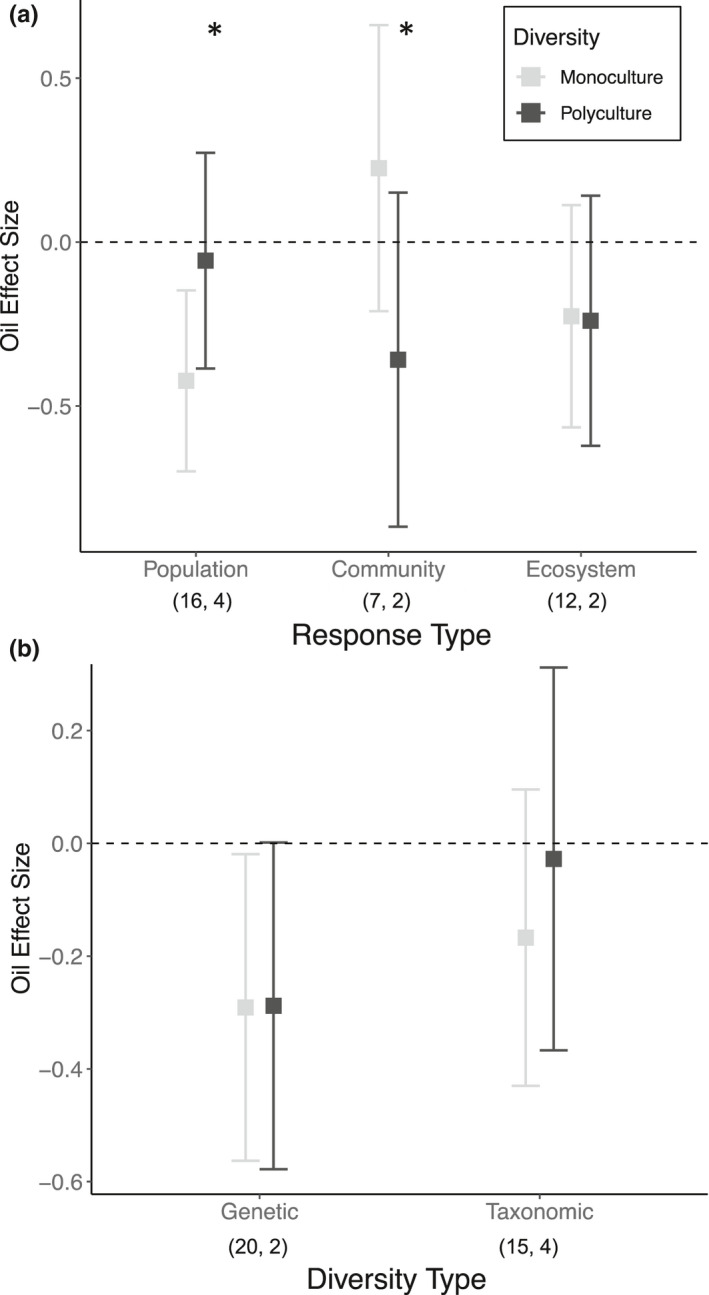
Estimated mean Hedge's *d* effect size of oiling ±95% confidence intervals in monoculture (light grey) and polycultures (black) across (a) response level (population, community, or ecosystem) and (b) diversity type (genetic or taxonomic). The numbers in parentheses (*n*, *k*) represent the number of effect sizes used in the models for both monoculture and polycultures within each response level or diversity type (*k*) and the number of independent studies from which those effect sizes were sourced from (*n*). A positive *d* indicates that oiling increased performance, while a negative *d* indicates that oiling reduced performance. 95% confidence intervals encompassing zero indicate no effect of oiling. * denotes significant different among monocultures and polycultures in oil effect within that response level (from post hoc linear contrast analyses) at level of *p* = .07

The presence of oil had an overall negative effect on the nearshore ecosystems tested in the mesocosm experiments (*d* = −0.21; 95% CI [−0.38; −0.045], Figure S1). Biodiversity impacted the response to oiling (*Q*_m_ = 6.18; *p* = 0.01), but the magnitude and direction varied among response types measured (biodiversity × response type: *Q*_m_ = 12.21; *p* = 0.02; Figure 1a). Polycultures reduced oiling effects on population‐level responses (estimated mean oiling effect [95% CI] = −0.06 [−0.39, 0.27) compared to monocultures (−0.42 [−0.70, −0.15); yet polycultures (−0.36 [−0.87, 0.15]) were more negatively impacted by oil than monocultures (0.23 [−0.21, 0.66]) when community‐level responses were measured (Figure 1a). In contrast, the type of biodiversity manipulated did not have a significant influence on the biodiversity effect (biodiversity x diversity type: *Q*_m_ = 2.05; *p* = .36; Figure 1b). However, genetic monocultures, but not polycultures, had a 95% CI that significantly differed from zero, suggestive of a more negative response to oiling with reduced genetic diversity.

### 3.3 B. Oil + Dispersant effects

**FIGURE 2:**
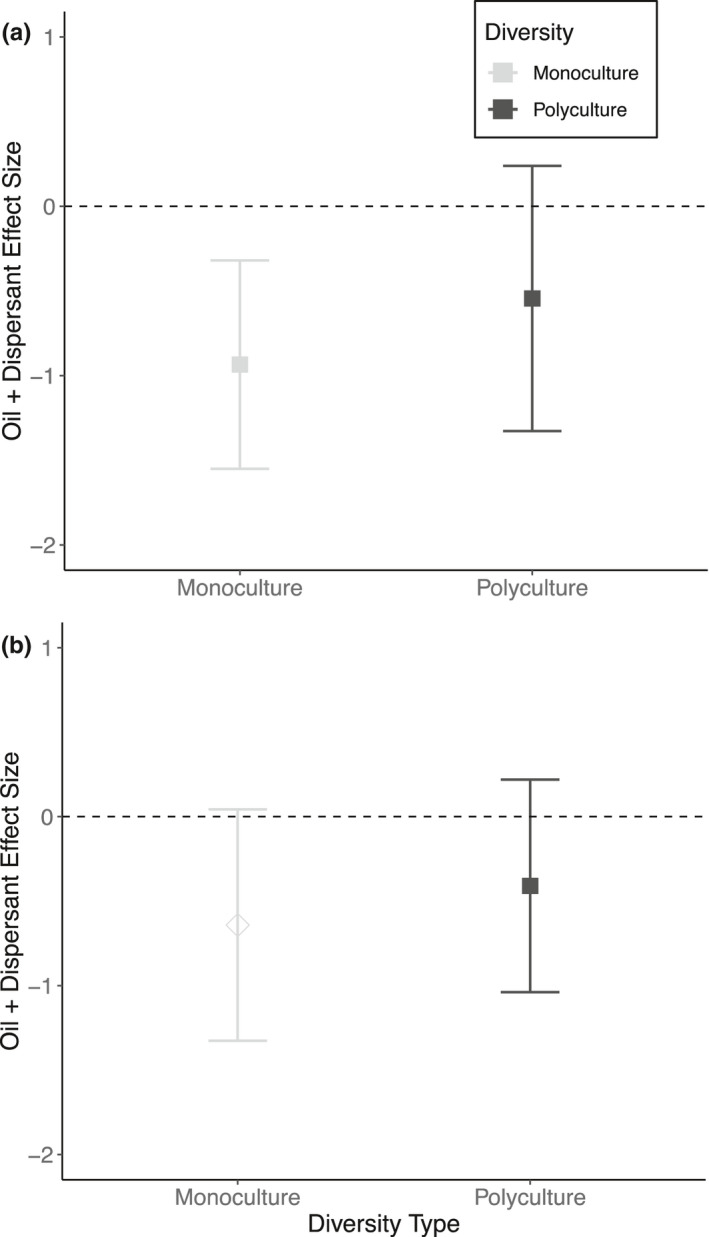
Estimated mean Hedge's *d* effect size of oil + dispersant ±95% confidence intervals between polyculture (black) and (a) average monoculture (grey, closed square) and (b) best monocultures (grey, open diamond). The number of effect sizes used in the models for both monoculture and polycultures was *k* = 5 for *n* = 2 independent studies from which those effect sizes were sourced from. A positive *d* indicates that oil + dispersant increased performance, while a negative *d* indicates that oil + dispersant reduced performance. 95% confidence intervals encompassing zero indicate no effect of oil + dispersant. Best monoculture was determined by the monoculture that was least impacted by oiling treatment

Similar to oil‐only effects, the cumulative effects of oil and dispersant in these experiments were also negative (*d* = −0.83; 95% CI [−1.50; −0.15], Figure S2). However, in contrast to oil‐only effects, there was no independent effect of biodiversity on the response to oil + dispersant (*Q*_m_ = 1.30; *p* = .25; Figure 2a).

### 3.4 Best monoculture versus average polyculture comparison

**FIGURE 3:**
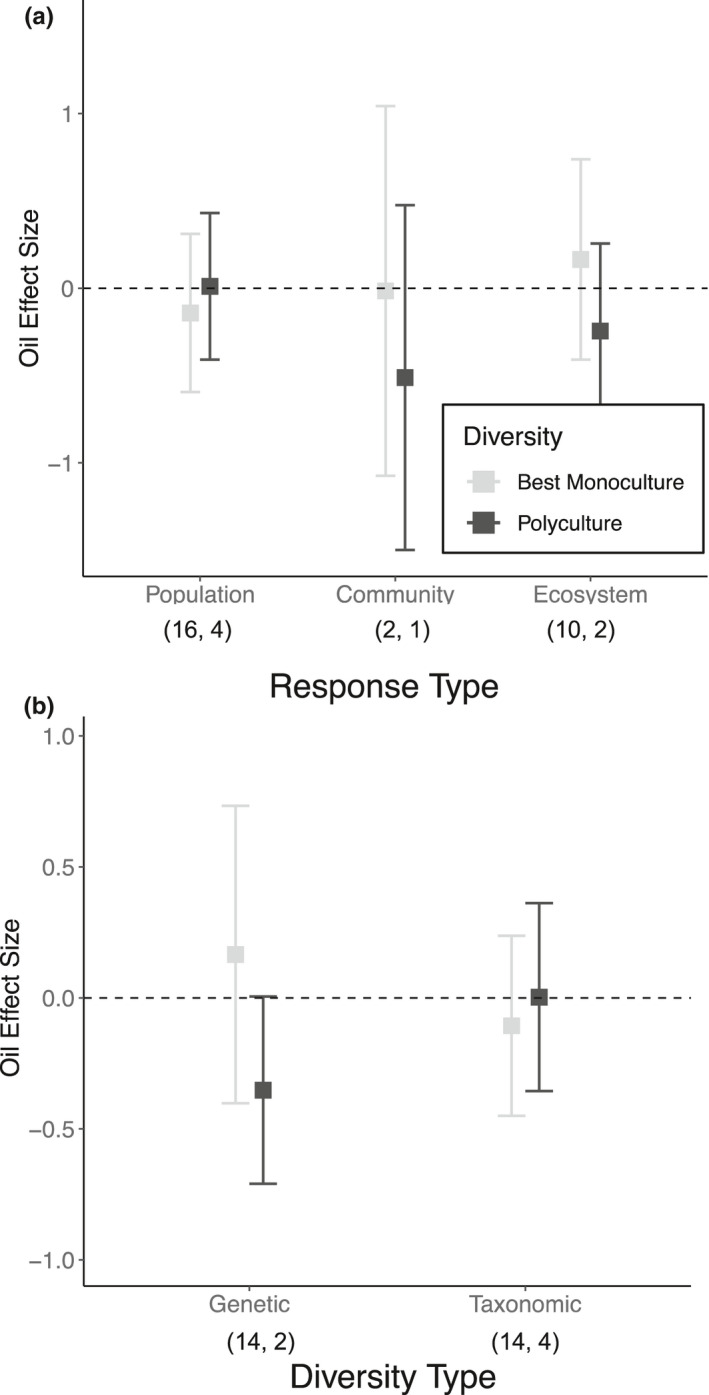
Estimated mean Hedge's *d* effect size of oiling ±95% confidence intervals in best monoculture (light gray) and polycultures (black) across (a) response level (population, community, or ecosystem) and (b) diversity type (genetic or taxonomic). The numbers in parentheses (*n*, *k*) represent the number of effect sizes used in the models for both monoculture and polycultures within each in response level or diversity (*k*) and the number of independent studies from which those effect sizes were sourced from (*n*). A positive *d* indicates that oiling increased performance, while a negative *d* indicates that oiling reduced performance. 95% confidence intervals encompassing zero indicate no effect of oiling. Best monoculture was determined by the monoculture that was least impacted by oiling treatment. Because we removed any experiment or response that did not replicate individual monocultures in this dataset, the average polyculture values are different from Figure 1

Interestingly, when we compared the results from the best monoculture (i.e., the taxon/genotype least affected by oiling) to those of polycultures, we found less striking and inconsistent patterns than the comparison of average responses. Biodiversity did influence oil‐only effects (*Q*_m_ = 4.26; *p* = .04). However, the direction of this effect depended on the type of diversity being manipulated (*Q*_m_ = 6.31; *p* = .04). While linear contrasts detected no significant differences within either diversity category, taxonomic polycultures (estimated mean oiling effect [95% CI] = 0.0027 [−0.36, 0.36]) had an oiling effect similar to the best monoculture (−0.11 [−0.45, 0.24]), whereas, in contrast, genetic polycultures (−0.35 [−0.71, 0.001]) tended to be more negatively affected than the best monoculture (0.17 [−0.40, 0.73]; Figure 3b). However, the interaction between biodiversity and the type of response measured was insignificant (*Q*_m_ = 6.25; *p* = .18, Figure 3a). Furthermore, there was no significant independent effect of biodiversity on oil + dispersant responses (*Q*_m_ = 0.27; *p* = .60; Figure 2b), consistent with the average monoculture results.

## 4 DISCUSSION

We add to the growing evidence that greater diversity can impact the response of marine ecosystem to disturbance (Salo & Gustafsson, 2016; Stachowicz et al., 2007; Worm et al., 2006). However, we also found that the magnitude and direction of diversity effects depended both on the properties of diversity and response metrics. Consistent with prior marine diversity–stability studies that focused on within‐trophic levels, we observed that polycultures tended to reduce the negative effects of oiling compared to the average response of single species or genotypes for population‐level responses in the nearshore communities tested (Figure 1a). Yet, community‐level responses to oiling were negatively impacted by diversity, highlighting the complexity that existed across different levels of biological organization. Furthermore, there tended to be stronger evidence for taxonomic diversity to lessen responses to oil disturbance than genetic diversity (Figure 1b). Further, our positive diversity effects on oil impacts are likely conservative due to both the relatively few experiments conducted, and the high variability in oil exposure levels tested across ACER studies. Meanwhile, diversity had no impact on the response of these coastal ecosystems to the combination of oil and dispersant within our limited dataset, suggesting that there is likely limited built‐in capacity or variation among and within marine species’ response to novel man‐made disturbances. Together, our results suggest that biodiversity may have contributed to the resilience of northern Gulf of Mexico ecosystems following *DwH* oil spill and, further, contributed to the variability found within and across *DwH* studies.

### 4.1 Across levels of biological organization

Positive diversity effects on oiling impacts were primarily evident when diversity was manipulated at the same trophic level for which the response was measured (i.e., population‐level responses; Figure 1a). This positive effect of biodiversity was likely underpinned by variation within and among northern Gulf of Mexico species’ responses to oiling (e.g. Fleeger et al., 2019; Lin et al., 2016; McCann et al., 2017; Pezeshki et al., 2000). Similarly, we observed that taxonomic and genotypic mixtures did not perform better than the ‘best’ monocultures (Figure 3), consistent with the lack of transgressive overyielding in previous marine biodiversity–ecosystem function syntheses (Gamfeldt et al., 2015; Stachowicz et al., 2007). This provides further evidence that identity effects contribute substantially to the observed diversity effect on population‐level responses, indicating that there are certain taxa and genotypes that are more capable of withstanding and/or recovering from oil exposure than others. Thus, greater biodiversity may be particularly important as it increases the likelihood that these individual taxa or genotypes are present (e.g. Boyer et al., 2009; Gamfledt & Kallstrom, 2007), in agreement with the insurance hypothesis (Yachi & Loreau, 1999). Complementarity (i.e., resource partitioning, facilitation) may also be contributing to the observed diversity effect, as previous experiments and models have illustrated that both can enhance the response of more diverse communities following a disturbance (Hughes & Stachowicz, 2011; Loreau & Mazancourt, 2013). However, we were unable to directly test for this within our dataset.

Even with the massive number of investigations following the DwH, there are still gaps in our understanding of how species‐specific responses and within‐species variation influence the effects of oiling. Future studies should attempt to determine the traits or mechanisms species employ that are correlated with this variation in response. For example, differences in plant life‐history traits (e.g., annuals or short‐life span, vegetative regeneration, widely dispersed seeds, and dormant seed bank) are well‐documented to allow species to persist or quickly recover from other types of disturbance (Lavorel et al., 1997; McIntyre et al., 1995; Sousa, 1980). Likewise, variation in motility and behavior may be another underappreciated mechanism allowing for variation in individuals’ responses to disturbance (e.g., Fodrie et al., 2014). The identification of relevant morphological, behavioral, and physiological traits would allow for both predictability of which individuals are more or less vulnerable to oil exposure as well as an assessment of whether these traits are similar to those that resist/recover from other types of disturbances. This would allow the identification of taxa and habitats that might be most vulnerable to future oil spills. We also observed that the identity of the least susceptible taxon or genotype (i.e., best monoculture) varied across performance metrics and environmental conditions within individual studies (see https://data.gulfresearchinitiative.org/data/R4.x262.000:0056 for identity of best monoculture). This indicates that even when identity effects are strong, more diverse systems increase the likelihood of containing individuals that can maximize multiple ecosystem functions simultaneously (Byrnes et al., 2014; Duffy et al., 2003; Gamfeldt & Kallstrom, 2007). Thus, from a management perspective, conserving and maintaining biodiversity can provide a benefit to marine ecosystems suffering disturbances by increasing the likelihood of preserving more resistant and resilient individuals.

Diversity effects on oiling impacts were not consistent across response types. Instead, we observed an increased benefit of monocultures relative to polycultures on community‐level responses, such as secondary production and predation rates, when exposed to oil. The relationship between diversity and stability across multiple trophic levels is complex with the theoretical possibility of multiple outcomes (Rooney & McCann, 2012; Thibault & Loreau, 2005). Along with variation in individuals’ responses to disturbance, community‐ and ecosystem‐level outcomes can depend on whether (a) multiple individuals perform a similar functions; (b) correlations exist between individuals’ response to a disturbance and the magnitude of their effect on the community process or ecosystem function; and (c) disturbance influences on species interactions (Oliver et al., 2015).

In the absence of disturbance, manipulating diversity within one trophic level can have cascading impacts on adjacent trophic levels (Stachowicz et al., 2007). For example, greater producer diversity can increase the abundance and diversity of consumers, predators, and decomposers (Duffy et al., 2003; Gustaffson & Bostrom, 2011); Zak et al., 2003), thereby enhancing secondary production due to increased resource partitioning (Duffy et al., 2003, 2007). While our analysis of oiling effect size (oil–non‐oiled response) did not directly compare monoculture and polyculture performance, a similar analysis of diversity effects under non‐oiled conditions (see Appendix S3) found that community‐level responses did exhibit higher levels of functioning in polycultures than monocultures. The reason for the absence of positive diversity effects on community‐level responses under oiling could be twofold. First, given that species vary in their responses to oiling, if individuals that are more negatively affected by oiling are also those that contribute more to the response function or if there is limited functional redundancy, this could reduce diversity effects under oil exposure. Alternatively, if all species are equally likely to be impacted via disturbance, greater production in the presence of higher diversity prior to disturbance could result in more biomass available for removal from a given perturbation, which could result in reduced resistance (i.e., greater biomass loss; Allison, 2004; Worm & Duffy, 2003). Therefore, it is possible that positive diversity effects on adjacent trophic levels in the absence of stressors may enhance loss following a disturbance.

Diversity effects arise from interactions among individuals and between individuals and their environments (Craven et al., 2016; Guerrero‐Ramirez & Eisenhauer, 2017). Because, environmental change—including disturbance—can modify the strength and direction of species interactions as well as how individuals’ response to a disturbance, diversity effects can vary across environmental conditions (Cardinale et al., 2000; Fridley, 2002; Steudel et al., 2012; Worm et al., 2002). For instance, variation in food or habitat preference mediates the strength of competitive interactions (Duffy et al., 2007), which can contribute to temporal stability across trophic levels (Gustaffon & Bostrom, 2011; Ramus & Long, 2016). Therefore, if oiling alters a species’ resource use or preferences, this, in turn, may alter the strength and direction of species interactions and ultimately drive variation in diversity effects across oil exposure. Thus, changes in resource availability and preference could underpin the variation in both microbial production and fish predation rates we observed between monocultures and polycultures (Figure S1). For instance, oil can promote and favor the abundance of certain microbes capable of hydrocarbon degradation (Bernhard et al., 2016; DeLaune & Wright 2011; Natter et al., 2012) and/or alter plant detrital inputs, an important resource that structures microbial communities (Waldrop et al., 2006). Together, these corresponding changes in resource availability could alter interactions among microbes (Kearns et al., 2016; She et al., 2018) and thus diversity effects. Similarly, oil can alter fish behavior (Fodrie et al., 2014; Martin, 2017) and changes in foraging rates or prey preferences (Tarnecki & Patterson, 2015) could reduce diversity effects, particularly if there was reduction in prey complementarity and/or increased competition among consumers. This combination of positive diversity effects under non‐oiled conditions, and alteration of resource specialization and species interactions with oiling, could underpin the negative or negligible diversity effects we observed at higher levels of biological organization.

### 4.2 Comparison of genetic and taxonomic diversity

Genetic diversity can have ecological effects comparable to those of taxonomic diversity (Bolnick et al., 2011; Des Roches et al., 2017; Hughes et al., 2008). Our meta‐analysis showed a trend of greater reduction of the negative effects of oiling with increasing taxonomic diversity but was more limited for genetic diversity. Similarly, data from two studies whose data were included here (Hughes et al., 2018; Schrandt et al., 2018) found little effect of genetic diversity on oiling impacts relative to other factors such as salinity and species composition. Yet, we also observed that only genetic monocultures showed a significantly negative effect of oiling (Figure 1b). Taxonomic monocultures likely contained some genetic variation, which suggests that there is likely to be within‐species variation that may buffer oiling effects. This suggests that while genetic variation may allow variation in response and possible adaptation to oiling, co‐occurring environmental stressors, and variation among species in their tolerance and susceptibility to oiling (Fleeger et al., 2019; Lin et al., 2016; Pezeshki et al., 2000) may play a larger role in marine ecosystems’ response following oil exposure.

### 4.3 Future directions

Heterogeneity in oil concentration, duration, and exposure methods (pulse vs. press exposure) among ACER experiments may have also contributed to the variability in our results. It is well documented that community resilience is in part determined by the size, frequency, and timing of a disturbance (Reice 1994). We were limited in our ability to test or account for this in our analyses, and variation in both experimental duration and frequency of sampling across studies limited our ability to separate the response to oiling into resistance to, and recovery from oil exposure. Because community recovery is based on postdisturbance biodiversity, composition, and abundance, resistance and resilience are inextricably linked in nature (Griffin et al., 2009; Oliver et al., 2015), suggesting this inability has few limitations with respect to natural recovery. It is an open question as to whether the results of these mesocosm experiments are supported by the natural recovery of coastal ecosystems post‐*DwH*, which is hard to evaluate given the lack of substantial prior *DwH* baseline data on composition, abundance, and diversity of many nearshore taxa (although see Murawski et al., 2020). However, we might expect that for highly mobile species such as fishes, spatial heterogeneity in oiling in the field and their ability to emigrate from disturbed areas, may limit the utility of mesocosm experiments to evaluate acute oiling effects. Given the unprecedented extent of field surveys and knowledge gained post‐*DwH*, researchers should be well equipped to address this gap in the event of future spills and any discrepancies among studies may highlight other factors, including migration, that can modify diversity effects.

Organisms used in the ACER experiments primarily came from habitats with little to no known prior exposure to oil. Adaptation to oil exposure may be possible (see Lee et al., 2017), particularly in regions of the Gulf of Mexico where natural seeps and small‐scale oiling events occur semifrequently (MacDonald et al., 2015; Pulster et al., 2020). Thus, it would be interesting to explore whether diversity is more or less important in reducing the impacts of oil disturbance to ecosystems such as those in south Louisiana, whose inhabitants likely have adapted to frequent oiling stress. This may help explain why prior studies have shown that chemically dispersed oil can have more deleterious effects than oil alone (e.g., bacteria: Radniecki et al., 2013, plankton species: Almeda et al., 2014, Beyer et al., 2016; and oysters: Laramore et al., 2014, Vignier et al., 2015; Figure S2). Chemical dispersants are only used in large oil spills, and therefore, less frequent exposure to dispersant may have led to limited variation in species’ responses and potential for adaptation. This lack of prior exposure to dispersants may also underpin the lack of diversity effects we observed in oil +dispersant effect size. Similarly, studies of drought tolerance in forests have illustrated that forest tree diversity enhances drought resistance but only in drought‐prone environments (Grossiord et al., 2014). Thus, prior exposure may be crucial in driving asynchrony in species–environment interactions. Together, with our findings, this highlights the need to understand the circumstances in which biodiversity can or cannot reduce the ecological effects of novel and extreme disturbance events, particularly in this time of rapid environmental change.

## CONFLICT OF INTEREST

The authors declare no conflict of interest.

## AUTHOR CONTRIBUTIONS

**Robyn Zerebecki:** Conceptualization (equal); Data curation (lead); Formal analysis (lead); Writing – original draft (lead); Writing – review & editing (equal). **Kenneth Heck:** Conceptualization (equal); Funding acquisition (lead); Writing – review & editing (equal). **John Valentine:** Conceptualization (equal); Funding acquisition (lead); Writing – review & editing (equal).

## Supporting information

Supplementary Material

## Data Availability

Data are publicly available through the Gulf of Mexico Research Initiative Information & Data Cooperative (GRIIDC) at https://data.gulfresearchinitiative.org (10.7266/YMJP922A). Data supporting the results are also available on Dryad (https://doi.org/10.5061/dryad.ht76hdrhm).
