# Supplementary material for: Biodiversity influences the effects of oil disturbance on coastal ecosystems
Source: Ecol Evol. 2022 Jan 24;12(1):e8532. doi: 10.1002/ece3.8532 (PMC8796919; doi:10.1002/ece3.8532)
Supplement: Supplementary file 1 — Supplementary Material [file ECE3-12-e8532-s001.docx]

Appendix S1: Additional Methods: data inclusion, exceptions and modifications to response metrics.

As described in the main text, most studies measured multiple response variables as well as responses at multiple time points that were not independent. To address these concerns, we first limited our comparisons to data collected at the final time point a response variable was measured. In most cases this was at the termination of the experiment, but in a few instances, we included metrics that were only measured at a single time point during the experiment. In addition, some metrics were integrated across the entire duration due the nature of the response (e.g., growth rate) or were averaged across time due to incomplete sampling at any one time point.

In cases where studies measured several related, but distinct responses, we chose to assess only a subset of these responses. First, we removed variables that were obviously highly correlated, keeping the variable we deemed most representative and inclusive (e.g., we used aboveground plant biomass but not highly correlated stem density or average stem height). Second, we relied on suggestions by ACER investigators to include the most appropriate and encompassing metrics for testing our hypothesis (e.g., denitrification potential instead of denitrification capacity) based on their expertise. However, in some cases, we retained multiple qualified responses, as each provided distinct information and there was no clear consensus on the most inclusive metric. These included: a) consumption rates of individual prey species (instead of total combined prey consumption); b) production of specific microbial groups (e.g. denitrifers); c) maximum depth and horizontal dispersion as measures of bioturbation; and d) flower and seed production as measures of plant reproductive output.

We also modified or calculated several response variables from raw data provided by ACER subgroups or deposited in GRIIDC. First, the wetland mesocosm experiment (Hughes et al. 2018) measured multiple metrics of plant production (e.g., stem density, height, etc.) so we used prior calculations (Hughes 2012, Osland et al. 2014) to obtain a single measure of aboveground biomass that included combined estimates of both target plant species when evaluating taxonomic diversity treatments. In contrast, to assess the effect of genetic diversity on oil effect sizes, we used the exact growth or biomass metrics that been obtained for *Spartina,* as this was the target species in these treatments. Second, we determined phytoplankton monoculture and mixture intrinsic growth rates using a logistic function in the ‘growth rates’ package in R to produce estimates of species’ biomass throughout each experimental trial. We estimated biomass of each species based on individual cell counts recorded at each time point and species-specific cell-to-volume (Menden-Deuer & Lessard 2000). In addition, several experiments, such as those on prey consumption and bioturbation, included no organism controls to assess whether oil affected prey survival and bioturbation in the absence of predators or bioturbators; we used individual t-tests to determine if there was a significant difference in the response of these controls across oil exposure. Because all tests were not significant (p > 0.17), signifying no effect of oil exposure on these responses in the absence of any diversity, we did not incorporate these corrections into our raw values. Therefore, while our mean values may be over or underestimations, there should be limited impact on our calculated effect sizes. Finally, two response variables we included, a positive ecosystem function was denoted by either lower (i.e. horizontal bioturbation; Dorgan et al. 2020) or more negative values (e.g., phosphate flux – negative denotes sediments acting as a sink; Marton & Roberts 2014). To account for this, we multiplied each mean response by -1 to allow smaller and more negative values to be a more positive response to oiling in the effect size calculations.

Lastly, our initial dataset had substantial heterogeneity (see main text), suggesting the inclusion of extreme effect size outliers, which can distort overall conclusions. Using the dmetar package in R, we found that the majority of these extreme effect sizes (> -4) came from a single experiment (oysters), which had measured both oyster survival and shell growth. Initially we only included survival in our dataset, because the method used to calculate shell growth also incorporated survival, meaning these were not independent responses. However, to deal with this high heterogeneity, we removed oyster survival and replaced it with shell growth, and this greatly improved heterogeneity (I^2^ = 42.2%) and the fit of linear models (i.e., normality and equal variances).

Appendix S2: Additional Methods & Results: linear mixed models

Along with the multilevel meta-regression described in the main text, we also conducted a linear mixed model using the lme4 (Bates et al. 2015) and lmerTest (Kuznetsova et al. 2017) packages that used Satterthwaite approximation for degrees of freedom to generate F and p-values. We used a similar model that include an independent effect of biodiversity, as well as the interaction between biodiversity and diversity category and the interaction between biodiversity and response type as fixed effects, with study (sub-group) identity and response variable nested in an experiment as random effects to account for the non-independence among effect sizes and the inverse variance as a weighting factor (see main text).

We observed similar patterns using linear mixed models compared to meta-regression for comparison of average monoculture and polyculture treatments on oil and oil + dispersant effect sizes. Biodiversity impacted the response to oiling (F_1,31_ = 6.24, p = 0.012). However, the magnitude and direction of this biodiversity effect on oil impacts varied across the type of measured response (Biodiversity x response type: F_4,34_ = 3.08, p = 0.03), but not with level of diversity manipulated (Biodiversity x diversity type: F_2,34_ =1.03, p = 0.37). In contrast, we observed no significant effect of biodiversity on oil + dispersant effects (F_1,4_ = 3.26, p =0.15).

When comparing best monocultures to average polycultures, we also found consistent patterns to meta-regression (main text) as when using linear mixed models. Biodiversity affected oiling effects (F_1,27_ = 4.28, p =0.05), and this tended to depend on type of diversity being manipulated (F_2,26_ = 3.18, p = 0.06). However, there was no significant interaction between biodiversity and response on oiling effects. In addition, biodiversity had no influence on oil + dispersant effects (F_1,5_ = 0.23, p =0.65).

Appendix S3: Comparison of monoculture and polyculture performance under non-oiled conditions: Methods, Results & Discussion

Biodiversity can have positive ecological effects on the structure and function of ecosystems in the absence of any disturbance (Loreau et al. 2001, Hooper et al. 2005, Stachowciz et al. 2007, Cardinale et al. 2012, Duffy et al. 2017). Therefore, to better understand some of our observed effects of diversity on realized oil impacts in nearshore coastal ecosystems, we evaluated whether diversity effects were present in the absence of oiling. To do so, we constructed a similar Hedge’s d effect size that compared the difference in response of monocultures to polycultures for each individual response in non-oiled treatments resulting in 33 individual effect sizes. This is slightly less than the number of oil effects sizes, but due to some experiments (i.e., plankton) having multiple oiling levels comparison. A positive d indicates that diversity increased performance, while negative d indicates that diversity reduced performance. A Hedge’s d with 95% confidence intervals that encompassed zero indicates that there was no difference between polyculture and monoculture on performance. We also performed a similar multilevel meta-regression as in the main text (see Methods for additional details) and included category of manipulated diversity (2 levels: genetic or taxonomic) and response type (3 levels: population, community or ecosystem) as fixed effects and response variable nested in an experiment as random effect in the model.

Response type significantly influenced the diversity effect (*Q*_m_ = 7.16; p = 0.03): diversity had a significant positive effect on community-level responses, but not population nor ecosystem-level functions (Fig S4). The type of diversity manipulated had a marginal impact on the diversity effect (*Q*_m_ = 1.89; p = 0.17) as taxonomic diversity tends to have more positive effect on ecological responses than genetic diversity (Fig S5).

Interestingly, we found a lack of diversity effect on population-level responses in the absence of oiling, but that diversity reduced the oiling effect on these responses. Similarly, prior eelgrass studies found no evidence of eelgrass diversity effect on primary production initially, however, following abiotic (e.g. thermal stress; Reusch et al. 2005) or biotic (e.g. grazing; Hughes & Stachowicz 2004) disturbance observed positive diversity effects. These studies lacked controls without disturbance to rigorously test this relationship, but, suggest that whether disturbance enhances or dampens diversity effects likely depends on whether it increases or decrease variation in response among and within individuals. Further, dampening of species and genotypes variation may also contribute to why community-level response showed that positive diversity effect in the absence of oiling, but that monocultures performed better with respect to oiling (see main text for further discussion).

**Figure S1.**  Forest plot of Hedge’s d Effect size of oiling with 95% confidence intervals for each response variable individually for polyculture and monoculture treatments ordered by study. Diversity category (genetic or taxon) and Response Type (Population, Community, Ecosystem) denote for each response variable. Red triangle denotes overall oil effect across all responses.

**Figure S2.** Forest plot of Hedge’s d Effect size of oil + dispersant with 95% confidence intervals for each response variable individually for polyculture and monoculture treatments ordered by study. Diversity category (genetic or taxon) and Response Type (Population, Community, Ecosystem) denote for each response variable. Red triangle denotes overall oil + dispersant effect across all responses.

Figure 3. Funnel plots of Oil- only (A,C) and Oil + Dispersant (B,D) datasets which included average monoculture and polycultures (A,B) or ‘best’ monoculture and average polyculture (C,D) effect sizes.

Figure S4. Diversity effect size (Hedge’s d) in the absence of oiling varied across response type. A positive d indicates that diversity increased performance, while a negative d indicates that diversity reduced performance. A Hedge’s d with 95% confidence intervals encompassing zero indicates no difference between monoculture and polyculture treatments.

Figure S5. Diversity effect in the absence of oiling varied across type of diversity manipulated. A positive d indicates that diversity increased performance, while a negative d indicates that diversity reduced performance. A Hedge’s d with 95% confidence intervals encompassing zero indicates no difference between monoculture and polyculture treatments.

**Literature Cited:**

Cardinale, B.J. et al. 2012. Biodiversity loss and its impact on humanity. Nature 486:59-67

Dorgan, K.M. et al. 2020. Investigating the sublethal effects of oil exposure on infaunal behavior, bioturbation, and sediment oxygen consumption. Mar Ecol Prog Ser 635:9-24

Duffy, J.E. et al. 2017.Biodiversity effects in the wild are common and as strong as key drivers of productivity. Nature 549:261-264

Higgins, J. P. T., and Thompson, S.G. 2002. Quantifying heterogeneity in meta-analysis. Statistics in Medicine 21:1539-1559

Hooper, D.U. et al. 2005. Effects of biodiversity on ecosystem functioning: A consensus of current knowledge. Ecological Monographs 75:3-35

Hughes, A.R. et al. 2018. Effects of oil exposure, plant species composition, and plant genotypic diversity on salt marsh and mangrove assemblages. Ecosphere 9:e02207.10.1002/ecs2.2207

Hughes, A.R. 2012. A neighboring plant species creates associational refuge for consumer and host. Ecology 93:1411-1420

Loreau, M. et al. 2001. Biodiversity and ecosystem functioning: Current knowledge and future challenges. Science 294:804-808

Marton, J.M., and Roberts, B.J. 2014. Spatial variability of phosphorous sorption dynamics in Louisiana salt marshes. J Geophy Res Biogeosci 119:451-465

Menden-Deuer, S., and Lessard, E.J. 2000. Carbon to volume relationship for dinoflagellates, diatoms, and other protist plankton. Limnol. Oceanogr 45:569-579

Osland, M.J. et al. 2014. Aboveground allometric models for freeze-affected black mangroves (*Avicennia germinans*): Equations for climate sensitive mangrove-marsh ecotone. PLoS One 9: e99604.

Stachowciz, J.J. et al. 2007.Understanding the effects of marine biodiversity on communities and ecosystems. Annu Rev Ecol Evol Syst 38:739-766.
